# Metagenomic Next-Generation Sequencing in the Diagnosis of HHV-1 Reactivation in a Critically Ill COVID-19 Patient: A Case Report

**DOI:** 10.3389/fmed.2021.715519

**Published:** 2021-10-04

**Authors:** Lei Shi, Han Xia, Matthew D. Moore, Chao Deng, Na Li, Hui Ren, Yunru Chen, Jinfeng Liu, Fenjing Du, Gezhi Zheng, Jing Li, Qunying Han, Wanhu Fan, Feng Ye, Shumei Lin, Zhengwen Liu, Hongjuan Liu, Yawen Wang, Jian Yang, Qingguang Liu, Yingren Zhao, Tianyan Chen

**Affiliations:** ^1^The First Affiliated Hospital of Xi'an Jiaotong University, Shaanxi, China; ^2^Hugobiotech Beijing Co., Ltd., Beijing, China; ^3^Department of Food Science, University of Massachusetts, Amherst, MA, United States

**Keywords:** COVID-19, HHV-1, metagenomic next-generation sequencing (mNGS), diagnosis, case report

## Abstract

**Background:** Secondary infections pose tremendous challenges in Coronavirus disease 2019 (COVID-19) treatment and are associated with higher mortality rates. Clinicians face of the challenge of diagnosing viral infections because of low sensitivity of available laboratory tests.

**Case Presentation:** A 66-year-old woman initially manifested fever and shortness of breath. She was diagnosed as critically ill with COVID-19 using quantitative reverse transcription PCR (RT-qPCR) and treated with antiviral therapy, ventilator and extracorporeal membrane oxygenation (ECMO). However, after the condition was relatively stabled for a few days, the patient deteriorated with fever, frequent cough, increased airway secretions, and increased exudative lesions in the lower right lung on chest X-rays, showing the possibility of a newly acquired infection, though sputum bacterial and fungal cultures and smears showed negative results. Using metagenomic next-generation sequencing (mNGS), we identified a reactivation of latent human herpes virus type 1 (HHV-1) in the respiratory tract, blood and gastrointestinal tract, resulting in a worsened clinical course in a critically ill COVID-19 patient on ECMO. Anti-HHV-1 therapy guided by these sequencing results effectively decreased HHV-1 levels, and improved the patient's clinical condition. After 49 days on ECMO and 67 days on the ventilator, the 66-year-old patient recovered and was discharged.

**Conclusions:** This case report demonstrates the potential value of mNGS for evidence-based treatment, and suggests that potential reactivation of latent viruses should be considered in critically ill COVID-19 patients.

## Background

Severe acute respiratory syndrome coronavirus 2 (SARS-CoV-2) is the seventh known coronavirus that can infect humans after HCoV-229E, HCoV-OC43, HCoV-NL63, HCoV-HKU1, SARS-CoV, and MERS-CoV. The Coronavirus disease 2019 (COVID-19) global pandemic caused by SARS-CoV-2 has affected 205,372,963 of the world's population, with 4,334,178 total deaths, as of August 13, 2021 (https://coronavirus.jhu.edu/map.html). The symptoms in the majority of patients infected with SARS-CoV-2 have been relatively mild, but 10–15% patients developed severe infections (1). Compared with patients with mild symptoms, the severe patients are older, with more comorbidities and lower lymphocyte counts (2, 3) and experience higher mortality. Secondary infections or co-infections, mainly by bacteria or viruses, occur in 10–30% of hospitalized COVID-19 patients (4), contributing to the high incidence of severe infection and mortality of COVID-19.

Human herpes virus type 1 (HHV-1) is a double stranded DNA virus that infects a large population worldwide. According to the conservative estimate, more than 90% of the human adult population has latently infected by HHV-1 globally (5–8). HHV-1 establishes latent infection within ganglial cells, of which the trigeminal ganglia are the most commonly affected. When the body's immunity weakens, HHV-1 can be reactivated and cause opportunistic infection. The mortality of influenza patients is increased with concomitant HHV-1 infection, but the pathogenesis remains unclear (9). Whether SARS-CoV-2 coinfected HSV may aggravate the condition of COVID-19 has not been reported yet. We present a case of COVID-19 infection, followed by HHV-1 reactivation that exacerbated the condition of COVID-19.

## Case Presentation

**Figure 1:**
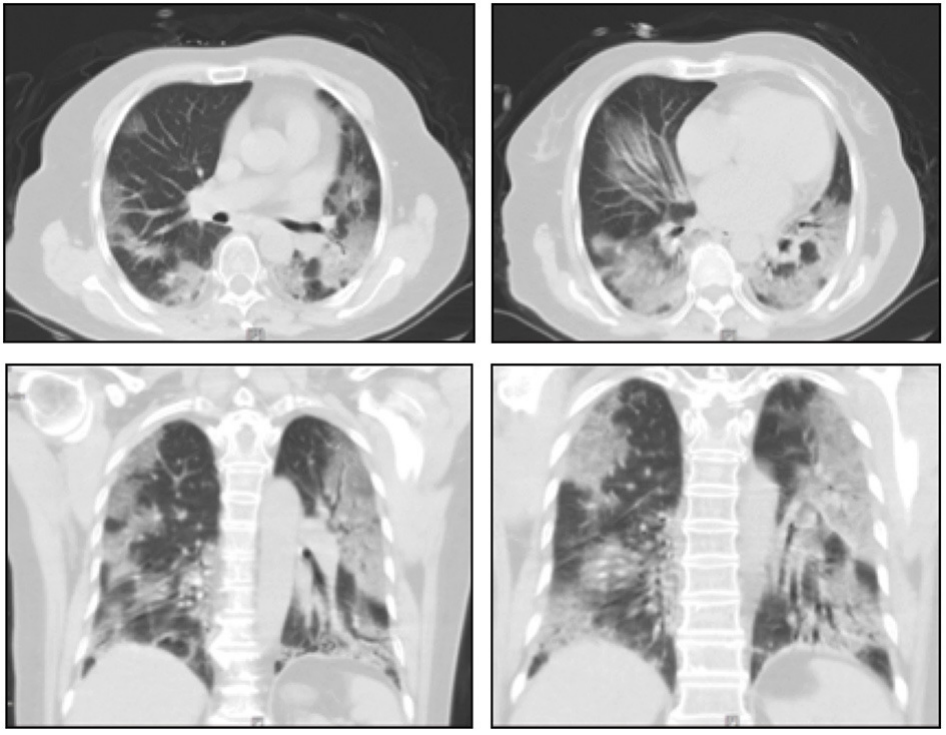
Representative images of the thoracic CT scans. Chest computed tomograms showing multifocal bilateral ground glass opacities with patchy consolidations, prominent in the peripheral distribution on day 4 after symptom onset.

A 66-year-old woman presented to the First Affiliated Hospital of Xi'an Jiaotong University on February 5, 2020, with a 4-day history of fever and shortness of breath. Apart from hypertension, the patient had no other underlying medical conditions. Laboratory tests were unremarkable except for a low lymphocyte count of 0.72 × 10^9^ cells/L (reference range 1.1–3.2 × 10^9^ cells/L) and high C-reactive protein of 71 mg/L (reference range 0-10 mg/L). The respiratory rate was 30 breaths/min, and oxygen saturation was 83% on ambient air. Chest computed tomography (CT) showed multifocal bilateral ground glass opacities (GGO) with patchy consolidations, prominent in the peripheral distribution and predilected to the posterior and lower lobes (Figure 1). Given a history of having contact with a confirmed COVID-19 patient and characteristic changes on CT imaging, a nasopharyngeal swab (NP) specimen was sent for SARS-CoV-2 testing by quantitative reverse transcription PCR (RT-qPCR). The swab registered as positive for SARS-CoV-2 while antigen tests for influenza A and B registered negative.

Upon admission, arbidol for antiviral therapy (0.2 g, oral, three times daily) and moxifloxacin for anti-bacterial therapy (400 mg, intravenous drips, once a day) were implemented to control patient's rapidly deteriorating condition. On Day 2 of hospitalization, the patient developed shortness of breath and chest tightness, and required supplemental oxygen *via* oxymask (oxygen flow at 10 L/min). Vitals included: respiratory rate 30–45 breaths per minute, oxygen saturation of 93–97% *via* pulse oximetry, and arterial blood gas PO_2_ 55 mmHg on mask oxygen at 10 L/min. Non-invasive ventilator-assisted ventilation was initiated.

**Figure 2:**
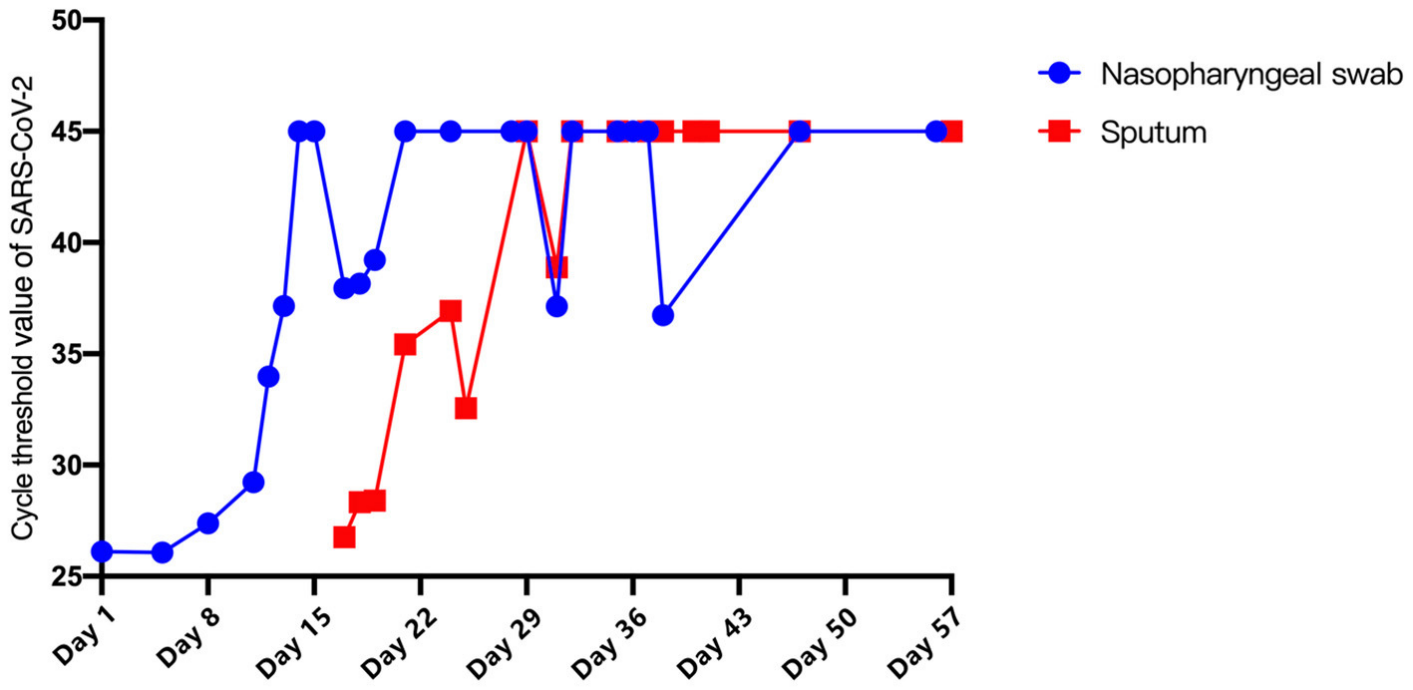
Cycle threshold value of SARS-CoV-2. Blue: Nasopharyngeal swab. Red: Sputum.

On Day 3, the patient's chest tightness and shortness of breath persisted. Chest X-rays indicated a superimposed pneumonic process. Hypoxemia showed no significant improvement after increasing PEEP and oxygen concentration. Based on the Extracorporeal Life Support Organization guidelines, extracorporeal membrane oxygenation (ECMO) (V-V mode) was initiated on Day 5. During this period, the patient's vital signs remained stable and SARS-CoV-2 viral copy number in NP samples continued to decrease (Figure 2).

On Day 14, the patient developed a frequent productive cough, and pulse oxygen saturation dropped to 88%, accompanied by increased heart rate (117 beats per minute), breathing rate (42 breaths per minute), and a temperature of 37.8°C. A small amount of light blood fluid was suctioned out of the endotracheal tube. Chest X-rays showed increased exudative lesions in the lower right lung. The white blood cell count increased to 18.39 × 10^9^ cells/L (reference range 3.5–9.5 × 10^9^ cells/L). Blood procalcitonin was negative, and sputum bacterial and fungal cultures and smears were negative, raising the possibility of a newly acquired infection, but with unknown etiology.

**Figure 3:**
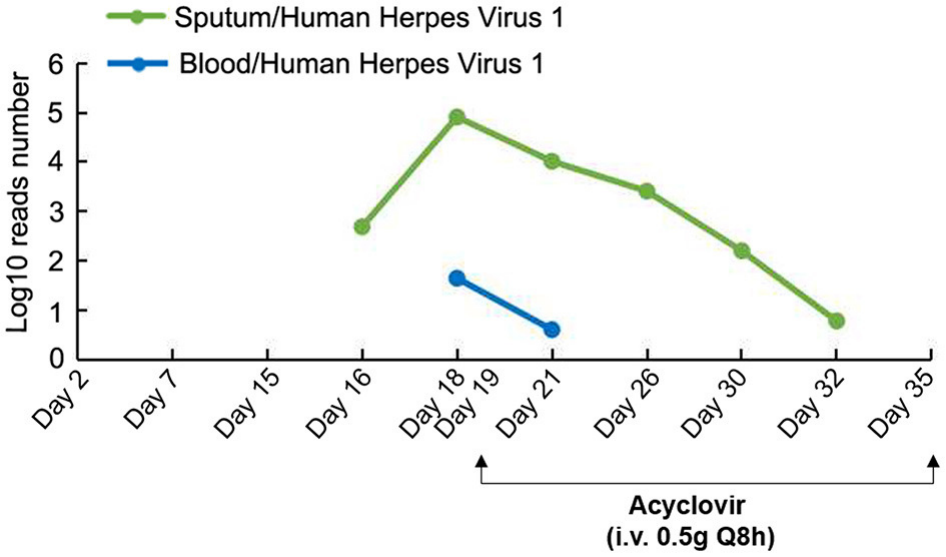
mNGS detection and anti HHV-1 therapy. Acyclovir: 0.5 g intravenous every 8 h from Day 19 to Day 35.

To further identify the presence of any newly acquired respiratory pathogens, PACEseq metagenomic next-generation sequencing (mNGS) (Hugobiotech, Beijing) of sputum was performed on Day 16 for pan-pathogen detection. The mNGS report came out on Day 18 (48 h after inspection) and showed that in addition to SARS-CoV-2 (9,266 reads/million), HHV-1 (484 reads/million) was predominant in the sputum. Then, mNGS re-examinations of samples including sputum, blood, NP, lung lavage, and stomach fluid were performed immediately on Day 18. The results showed the amount of HHV-1 in sputum increased to 81,098 reads per million (RPM). HHV-1 was also detected in blood (44 RPM), NP (89,833 RPM), lung lavage (8 RPM) and stomach fluid (46 RPM), suggesting a latent reactivation of HHV-1 (Figure 3). Guided by these sequencing results, an anti HHV-1 agent, acyclovir (0.5 g, intravenous every 8 h), was initiated. After 14 days of anti-HHV-1 therapy, the number of HHV-1 reads decreased and reached to single digit level of RPM. The patient's clinical condition improved, with normal body temperature, heart rate (62 beats per minute), breathing rate (15 breaths per minute), oxygen saturation of 97–100%, and white blood cell count (8.97 × 10^9^ cells/L). Chest X-ray showed the lesions in the lower right lung were no longer visible.

In subsequent days, the patient's clinical symptoms continued to improve, SARS-CoV-2 tests in sputum and NP maintained negative, and mNGS revealed no HHV-1 reads in sputum and blood. After 49 days on ECMO (February 9th-March 28th) and 67 days on the ventilator (February 6th-April 13th), the patient recovered and was finally discharged.

## Discussion and Conclusions

To the best of our knowledge, this is the first report describing re-activation of latent HHV-1 in a critically ill COVID-19 patient with prolonged ECMO support. HHV-1 is a double stranded DNA virus of genus Simplexvirus, family Herpesviridae. HHV-1 commonly resides in sensory ganglia in latency. A variety of stimuli such as stress, fever and immune suppression can trigger lytic replication and result in production of infectious virus (10). In an immune competent patient, HHV-1 infection is mild and self-limiting. However, HHV-1 infection can be severe and prolonged in immunocompromised individuals without treatment, leading to significant morbidity and mortality (11). In this report, the lymphocyte count of the patient decreased significantly when admitted to the hospital for COVID-19 and during hospitalization. Lymphopenia is common in severe COVID-19 patients (12). ECMO therapy is also associated with a decrease of lymphocyte count and functionality (13). Interestingly, CD8^+^ T cells play an essential role in preventing HHV-1 reactivation (14). The lower levels of lymphocytes in the reported patient might have permitted or promoted the reactivation of HHV-1 virus (15). Anti-HHV-1 therapy using acyclovir decreased HHV-1 load and alleviated the patient's clinical symptoms, indicating the significance of HHV-1 infection in the COVID-19 patient. We don't have direct evidence of HHV-1 pneumonia, but the clinical scenario (mNGS detection and the effect of anti HHV-1 treatment) is highly suggestive of causation.

Our report calls for awareness of latent virus reactivation in severe COVID-19 patients, particularly when infectious agents are unknown. Identification of latent virus activation becomes a challenging task for the routine diagnostic tests if the causative agents are unknown. Our report utilized mNGS technology, which successfully detected HHV-1 DNA in multiple body sites, and was valuable in monitoring the dynamics of viral loads over treatment course.

mNGS is a new pan-pathogen detection technology, which can theoretically detect all known pathogens including bacteria, viruses, fungi, and parasites in one run, with unbiased results. Compared to the conventional diagnostic methods, mNGS has a higher sensitivity and specificity and costs less time. In this case report, mNGS successfully detected HHV-1 in different types of samples, including sputum, blood, NP, lung lavage, and stomach fluid within 2 days, indicating significant advantages in the diagnosis of pathogens, which contributes to a timely treatment to the patient.

In conclusion, we present a case of COVID-19 with HHV-1, followed by HHV-1 reactivation that exacerbated the condition of COVID-19. mNGS was used for the rapid clinical diagnosis of the pathogen, demonstrating the potential value of metagenomics for evidence-based treatment. This case report suggests that potential reactivation of latent viruses should be considered in critically ill COVID-19 patients.

## Data Availability Statement

The original contributions presented in the study are included in the article/Supplementary Material, further inquiries can be directed to the corresponding author/s.

## Ethics Statement

The study was approved by the Ethics Review Committee of the First Affiliated Hospital of Xi'an Jiaotong University. The patients/participants provided their written informed consent to participate in this study. Written informed consent was obtained from the individual(s) for the publication of any potentially identifiable images or data included in this article.

## Author Contributions

LS, QL, YZ, and TC designed research. LS, HX, FD, GZ, JL, QH, WF, FY, SL, ZL, HL, YW, JY, and TC analyzed data. LS, HX, MM, CD, NL, HR, YC, JLiu, and TC wrote the paper. All authors have read and approved the manuscript.

## Funding

This study was funded by the Key R&D plan of Shaanxi Province, China (number 2020ZDXM-SF-004). The funding body had no role in study design; collection, analysis, and interpretation of data; writing of the report; or the decision to submit the paper for publication.

## Conflict of Interest

HX is employed by Hugobiotech Beijing Co., Ltd. The remaining authors declare that the research was conducted in the absence of any commercial or financial relationships that could be construed as a potential conflict of interest.

## Publisher's Note

All claims expressed in this article are solely those of the authors and do not necessarily represent those of their affiliated organizations, or those of the publisher, the editors and the reviewers. Any product that may be evaluated in this article, or claim that may be made by its manufacturer, is not guaranteed or endorsed by the publisher.

## Abbreviations

COVID-19: Coronavirus disease 2019
HHV-1: Human herpes virus type 1
SARS-CoV-2: Severe acute respiratory syndrome coronavirus 2
CT: Computed tomography
NP: nasopharyngeal swab
RT-qPCR: Quantitative reverse transcription PCR
ECMO: Extracorporeal membrane oxygenation
mNGS: Metagenomic next-generation sequencing.
