# Supplementary figures and images for: Metagenomic Next-Generation Sequencing in the Diagnosis of HHV-1 Reactivation in a Critically Ill COVID-19 Patient: A Case Report
Source: Front Med (Lausanne). 2021 Oct 4;8:715519. doi: 10.3389/fmed.2021.715519 (PMC8520926; doi:10.3389/fmed.2021.715519)

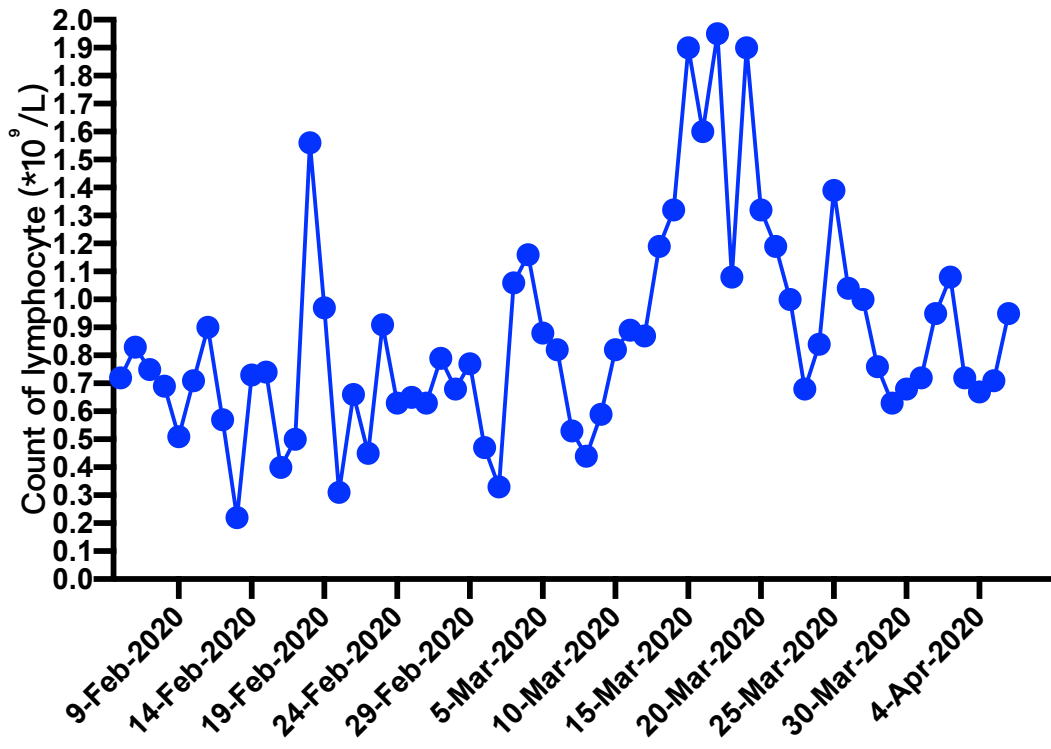

Supplement: Supplementary file 15 [file Data_Sheet_1.PDF]

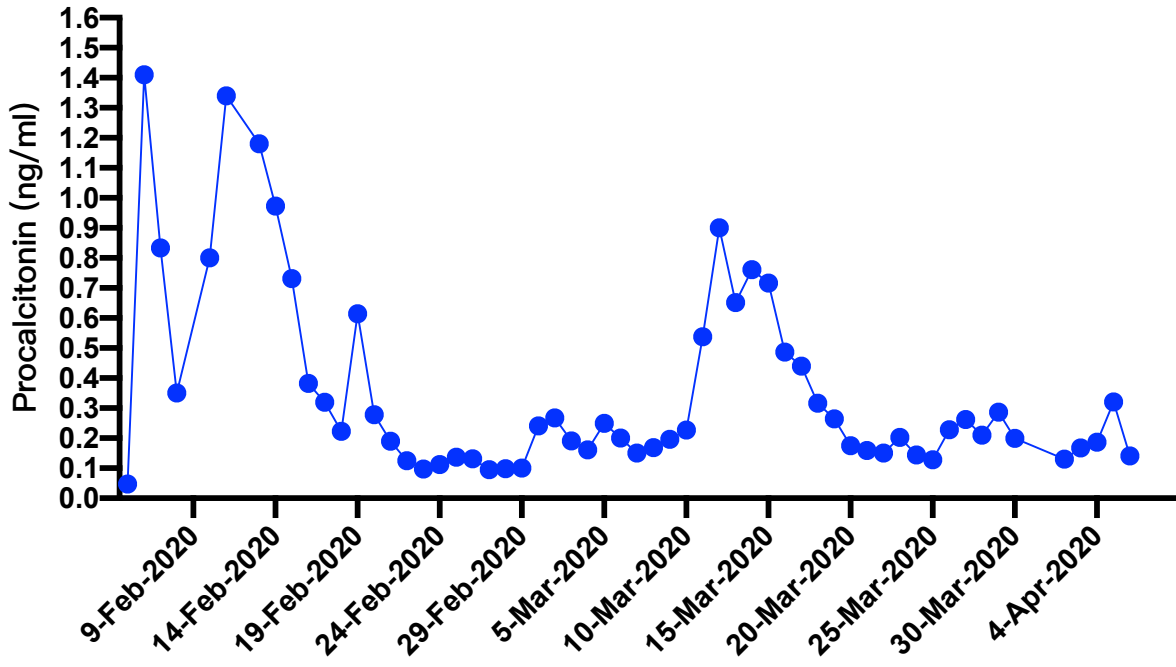

Supplement: Supplementary file 16 [file Data_Sheet_2.PDF]

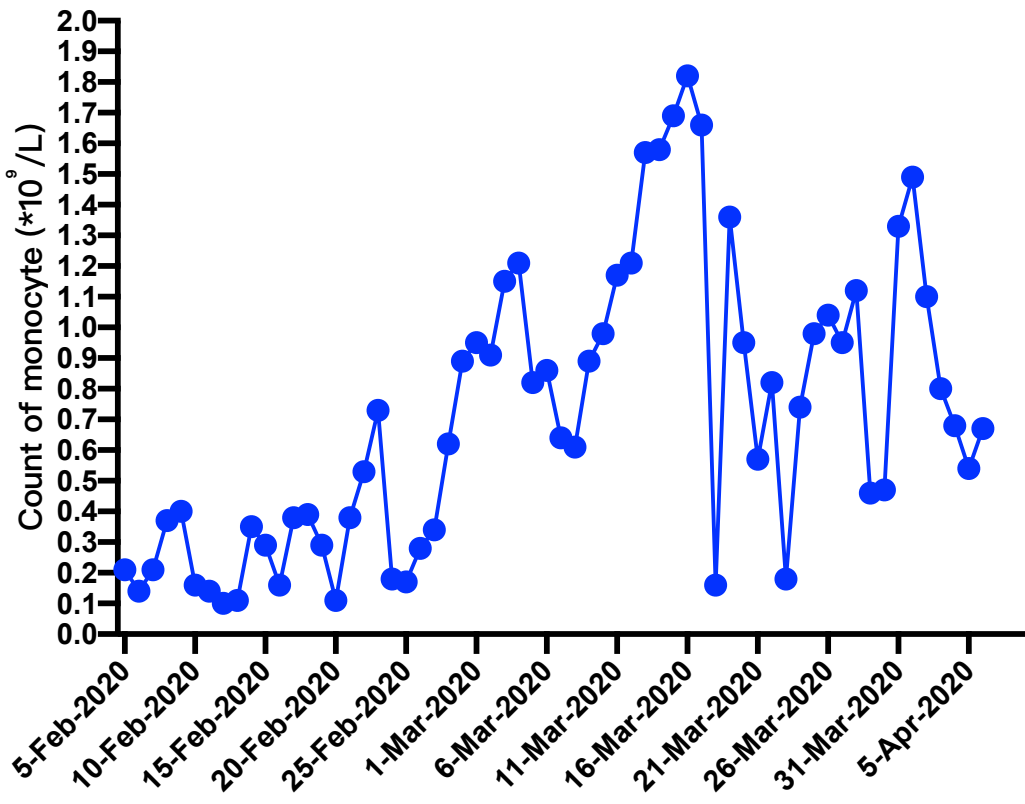

Supplement: Supplementary file 17 [file Data_Sheet_3.PDF]

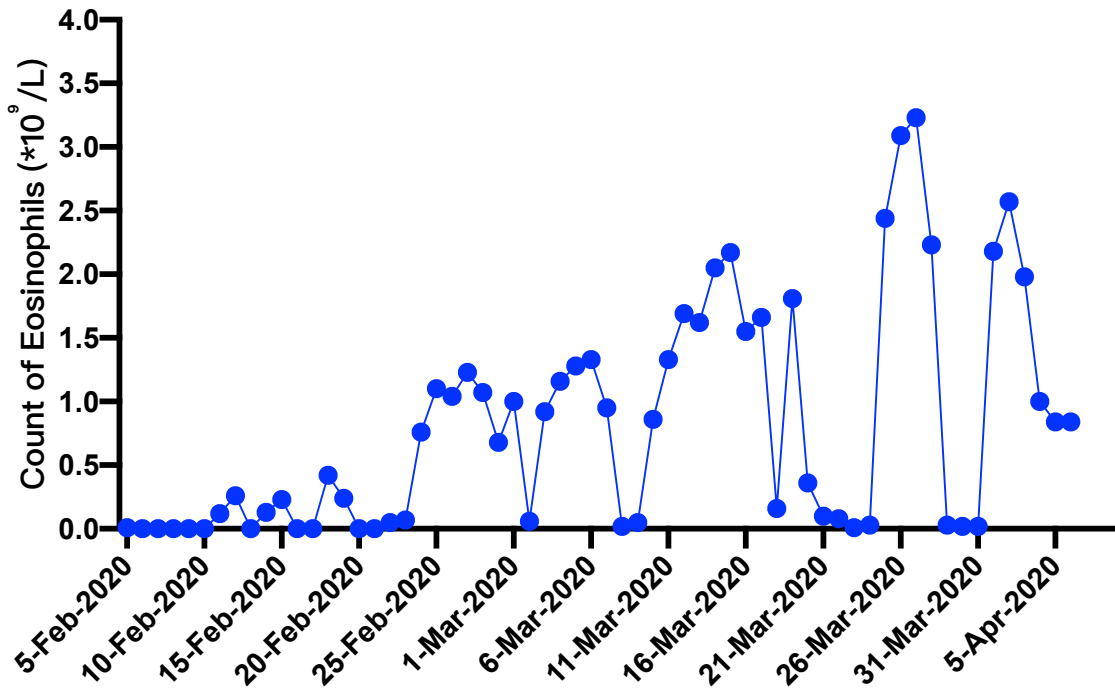

Supplement: Supplementary file 18 [file Data_Sheet_4.PDF]

报告编号：0111179692


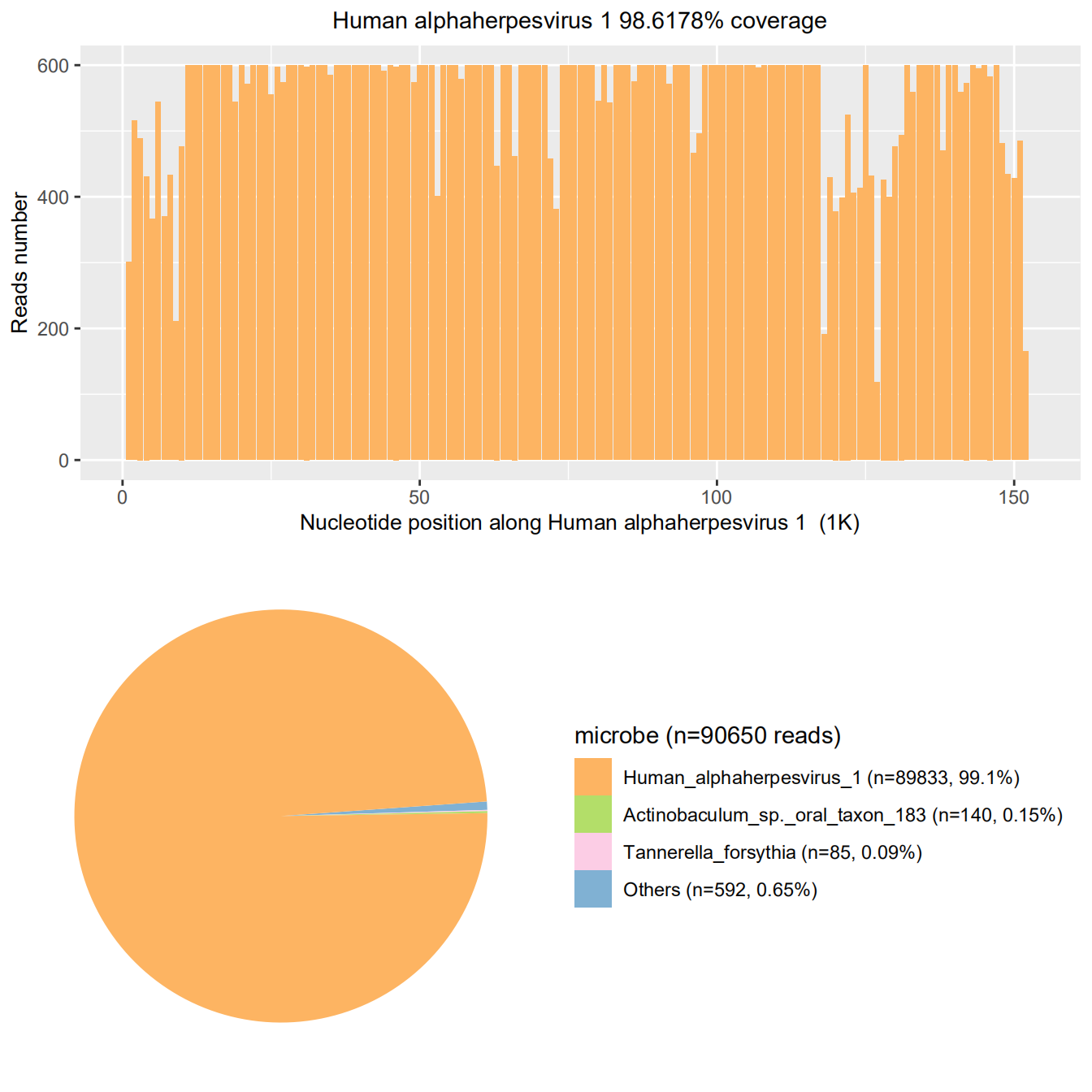


报告编号：0711179088


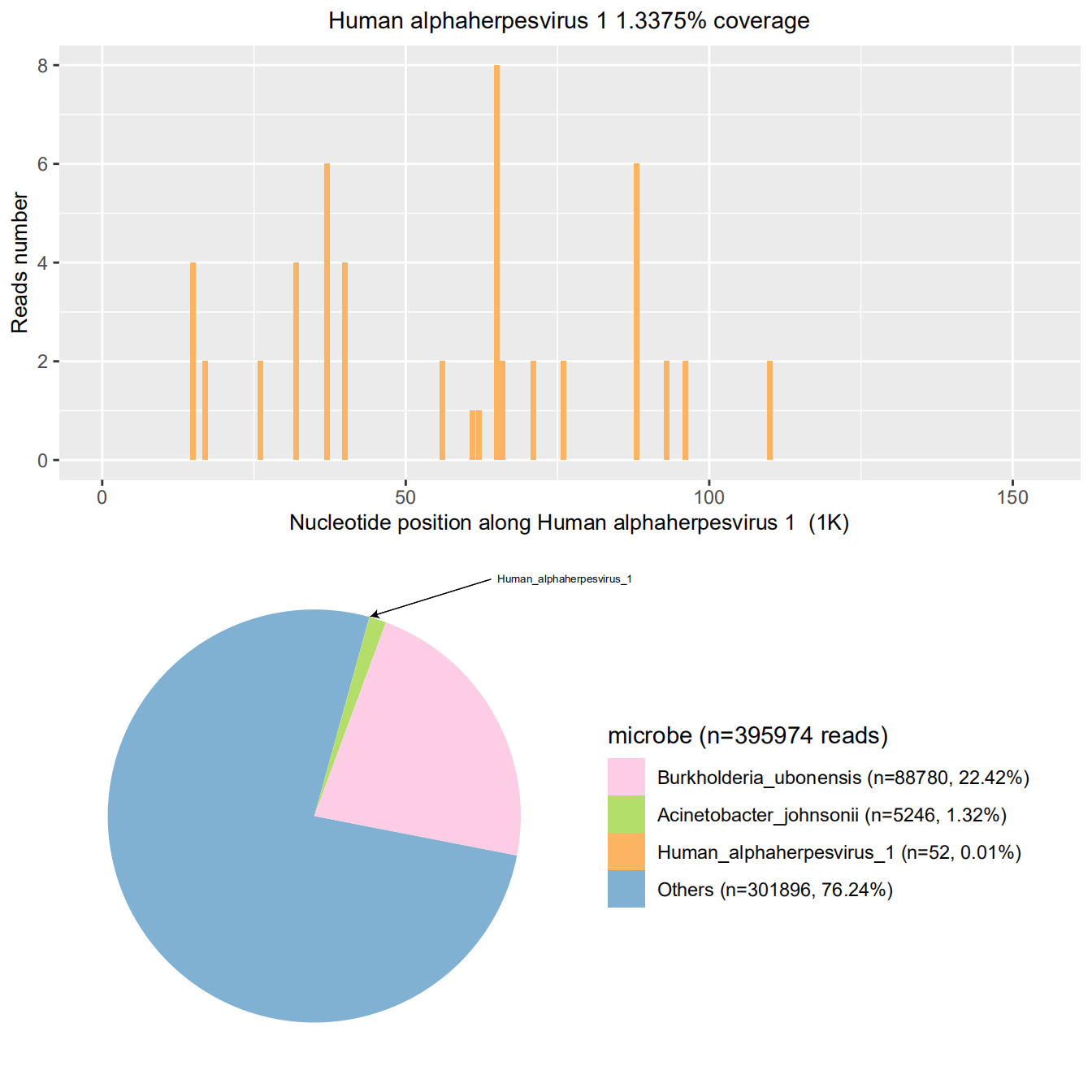


报告编号：0911179054


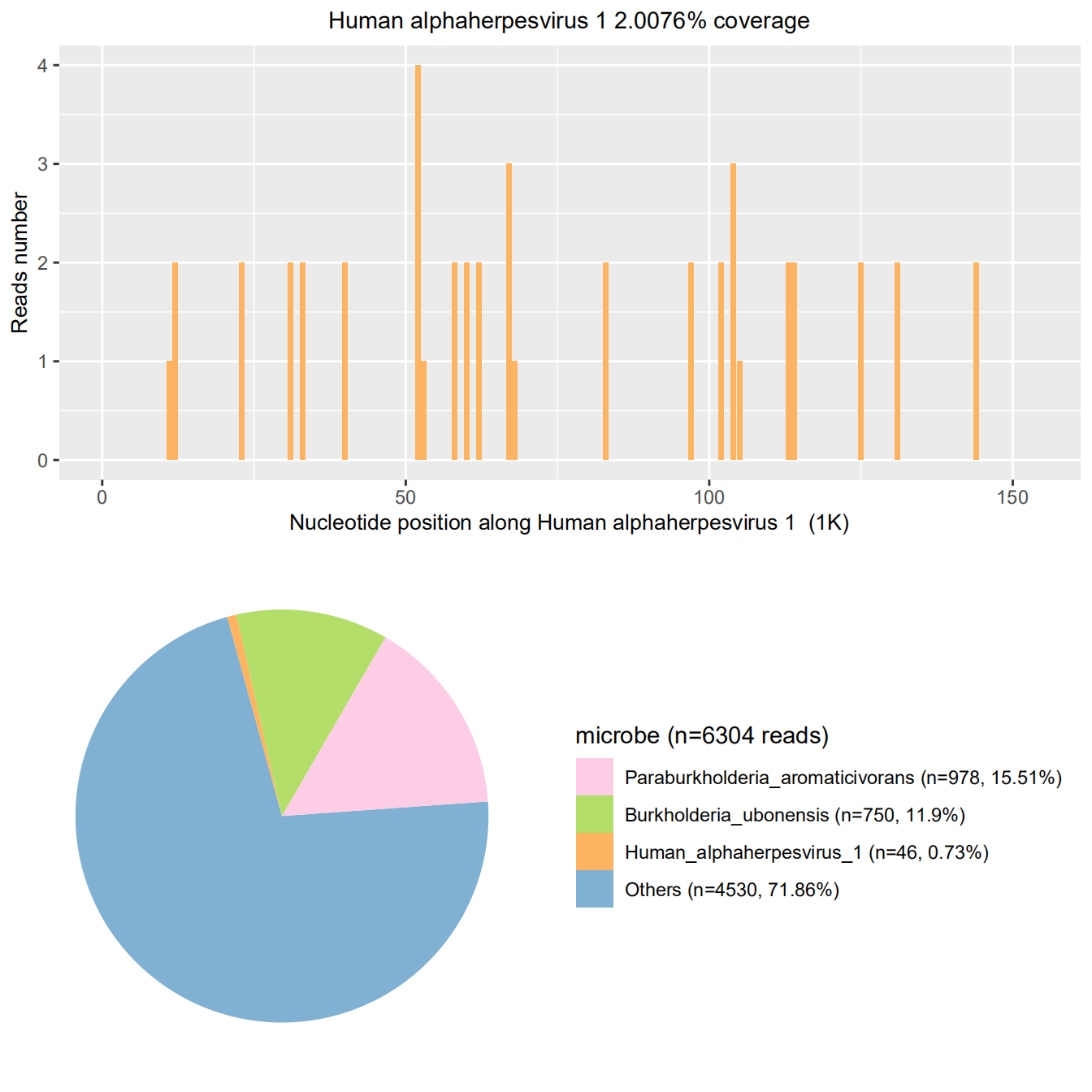


报告编号：2211178961


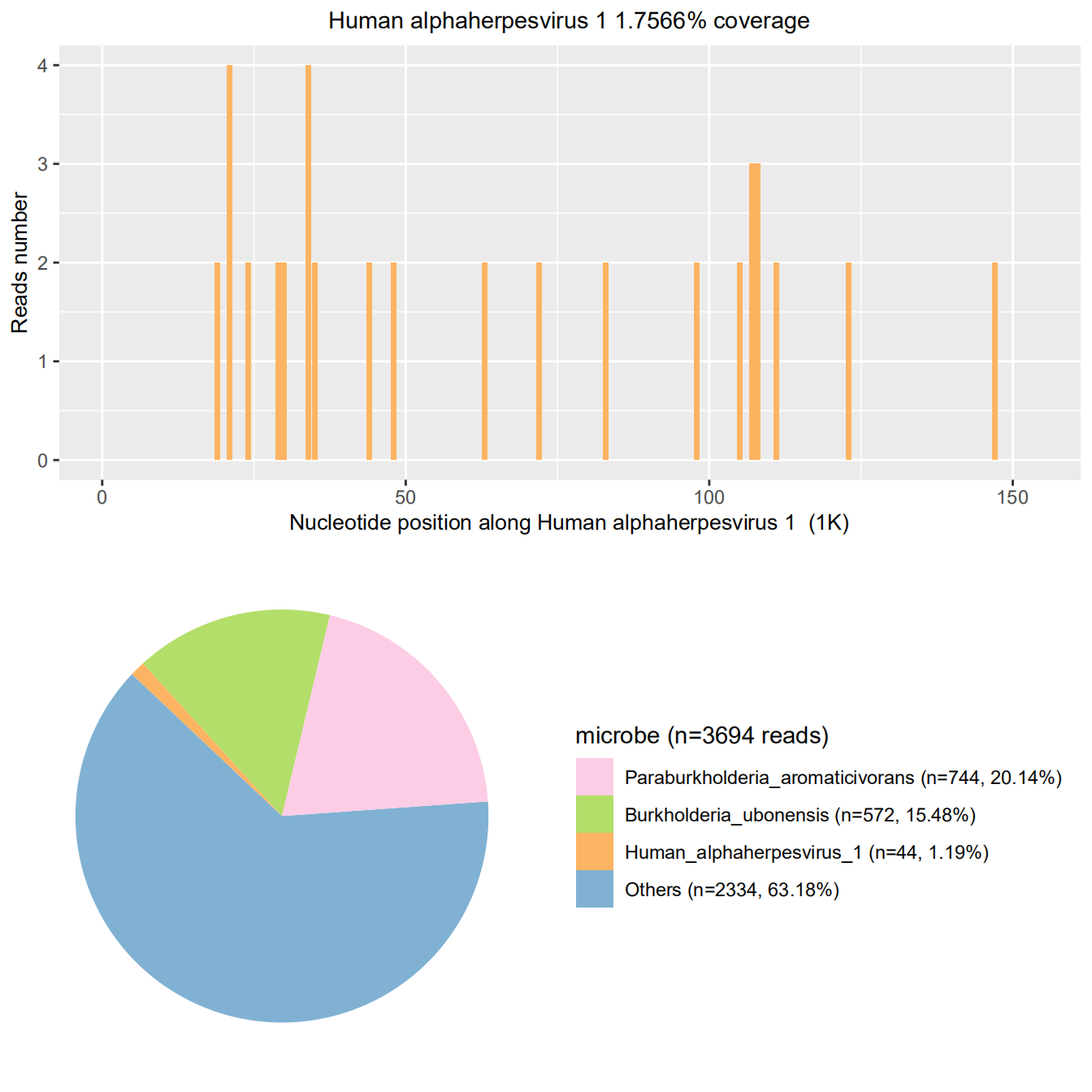


报告编号：2611179181


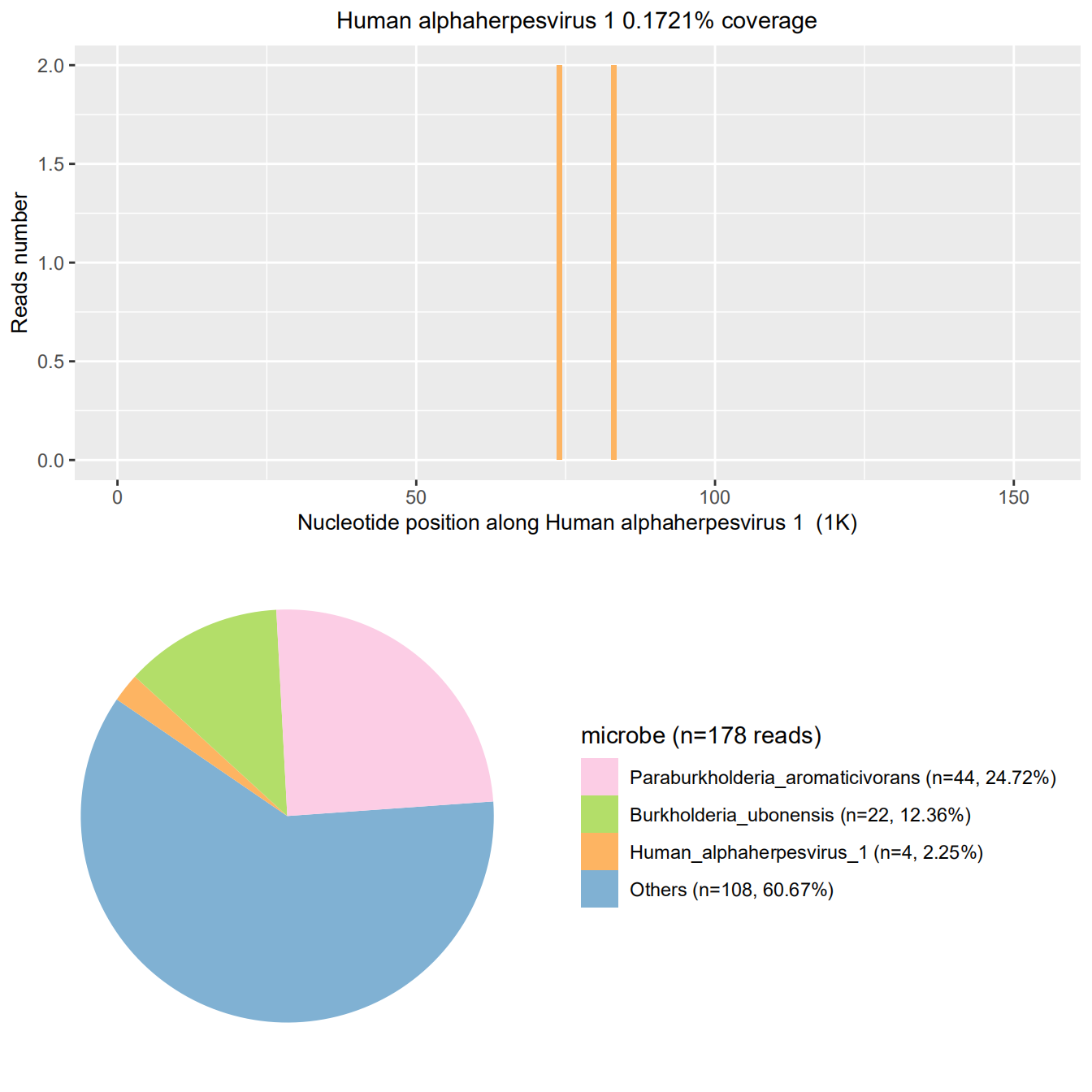


报告编号：3611181896


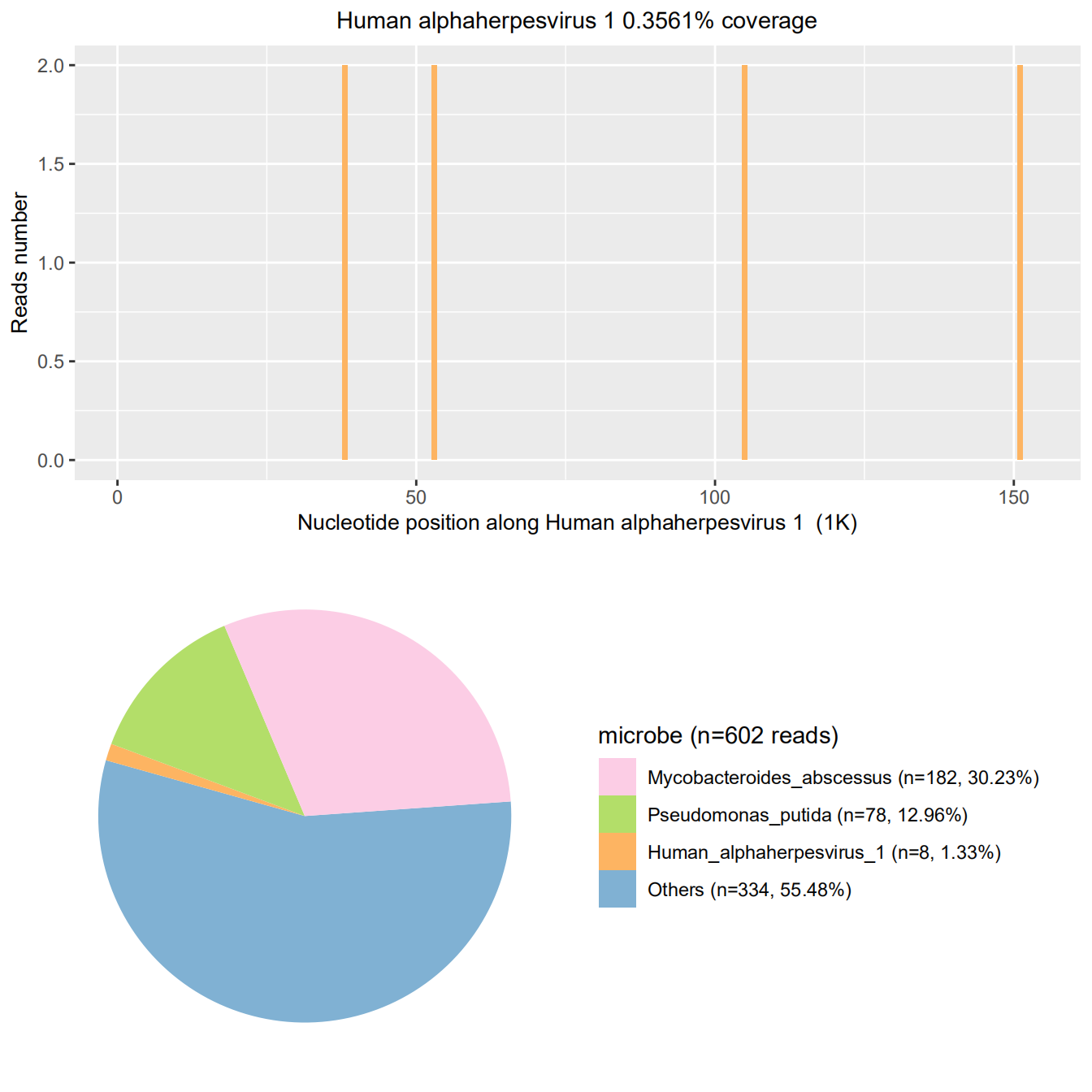


报告编号：6111179402


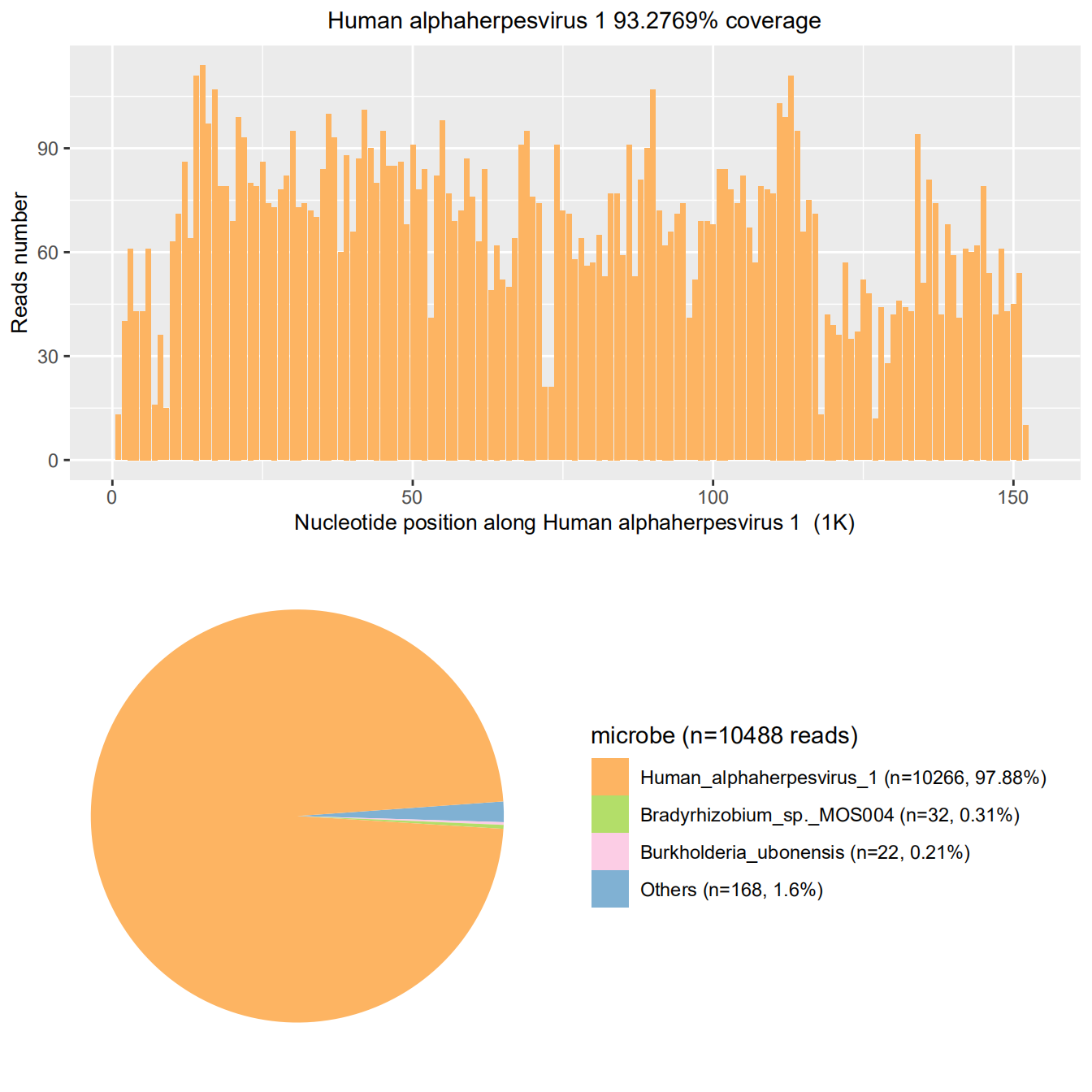


报告编号：6311181199


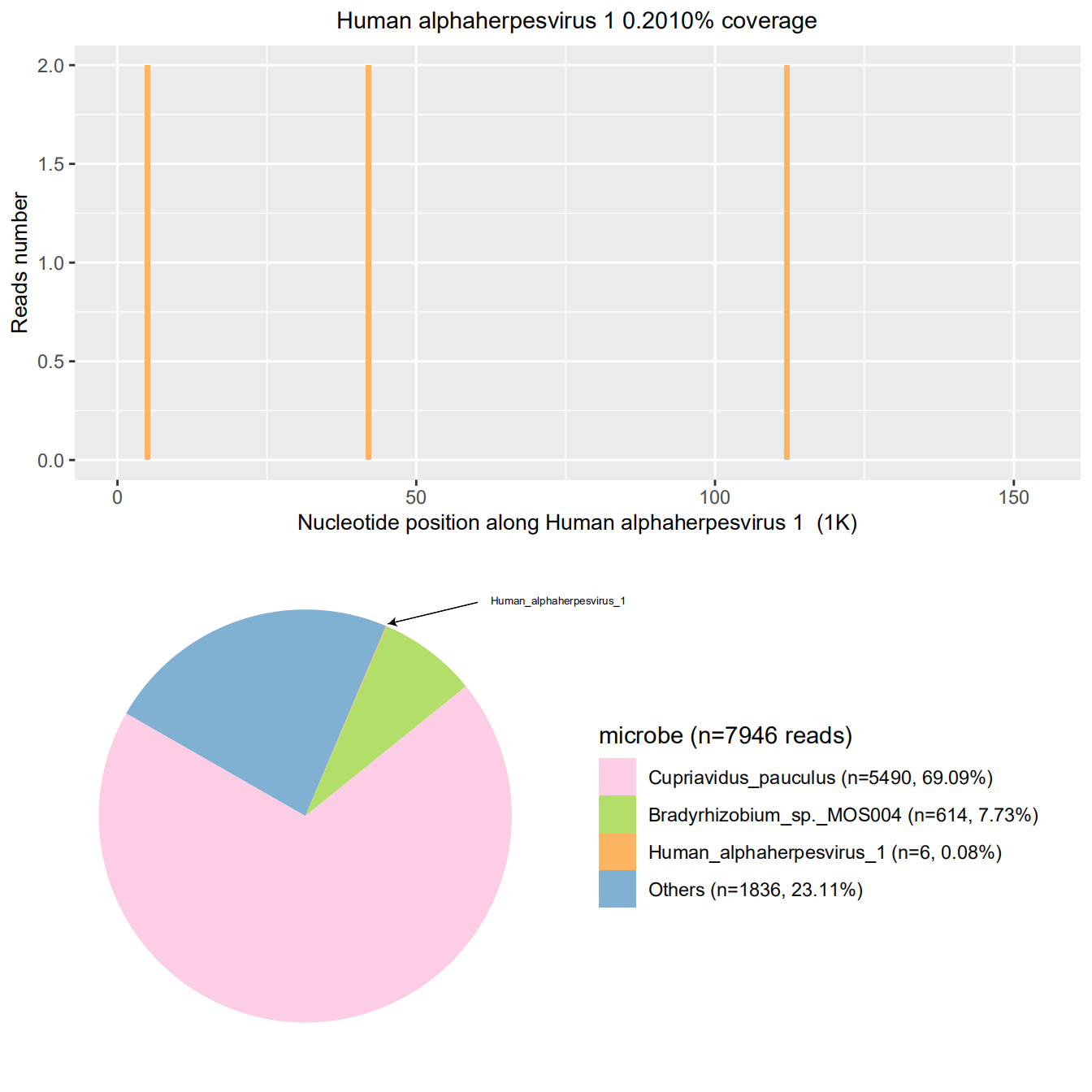


报告编号：6811178748


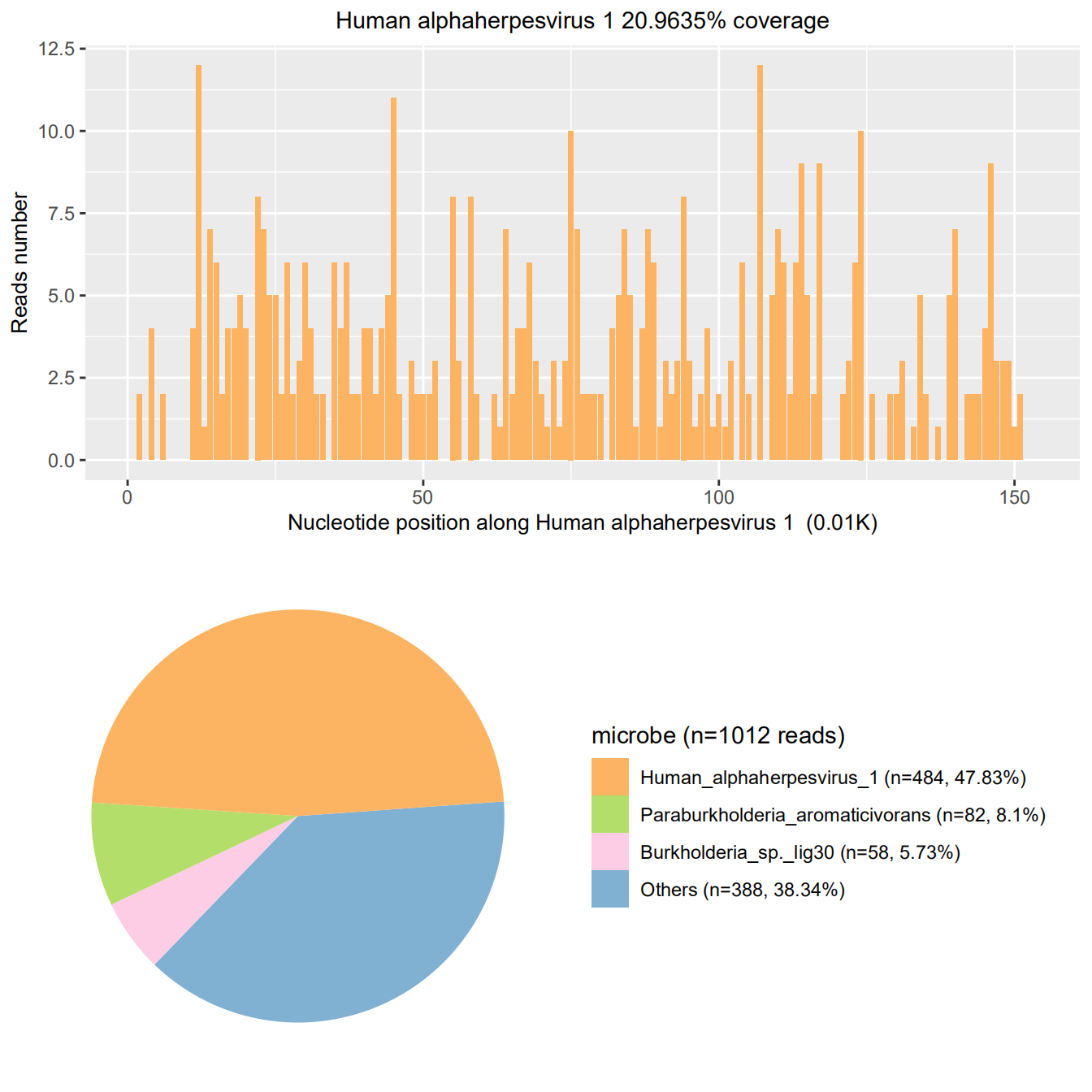


报告编号：6911170622


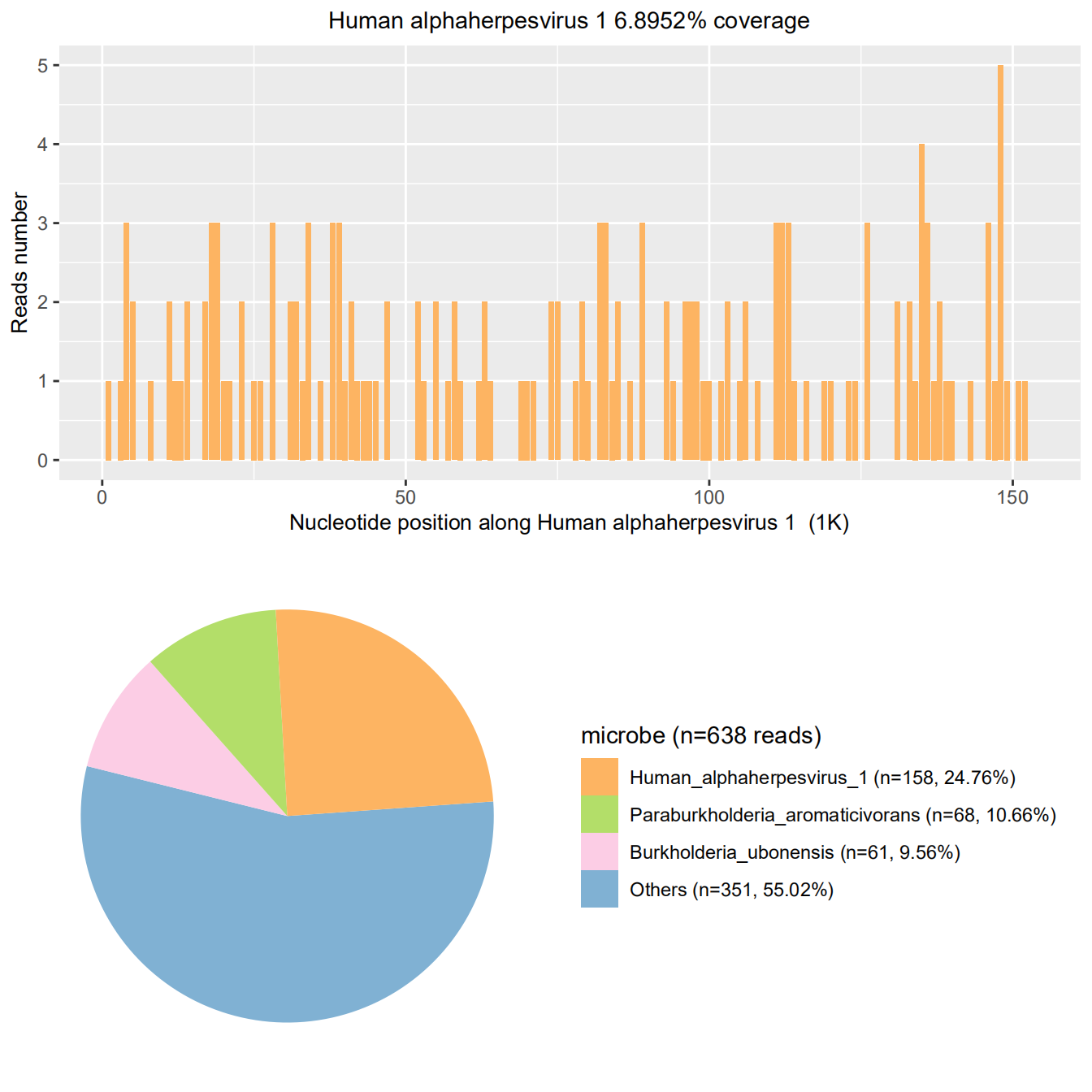


报告编号：6911178972


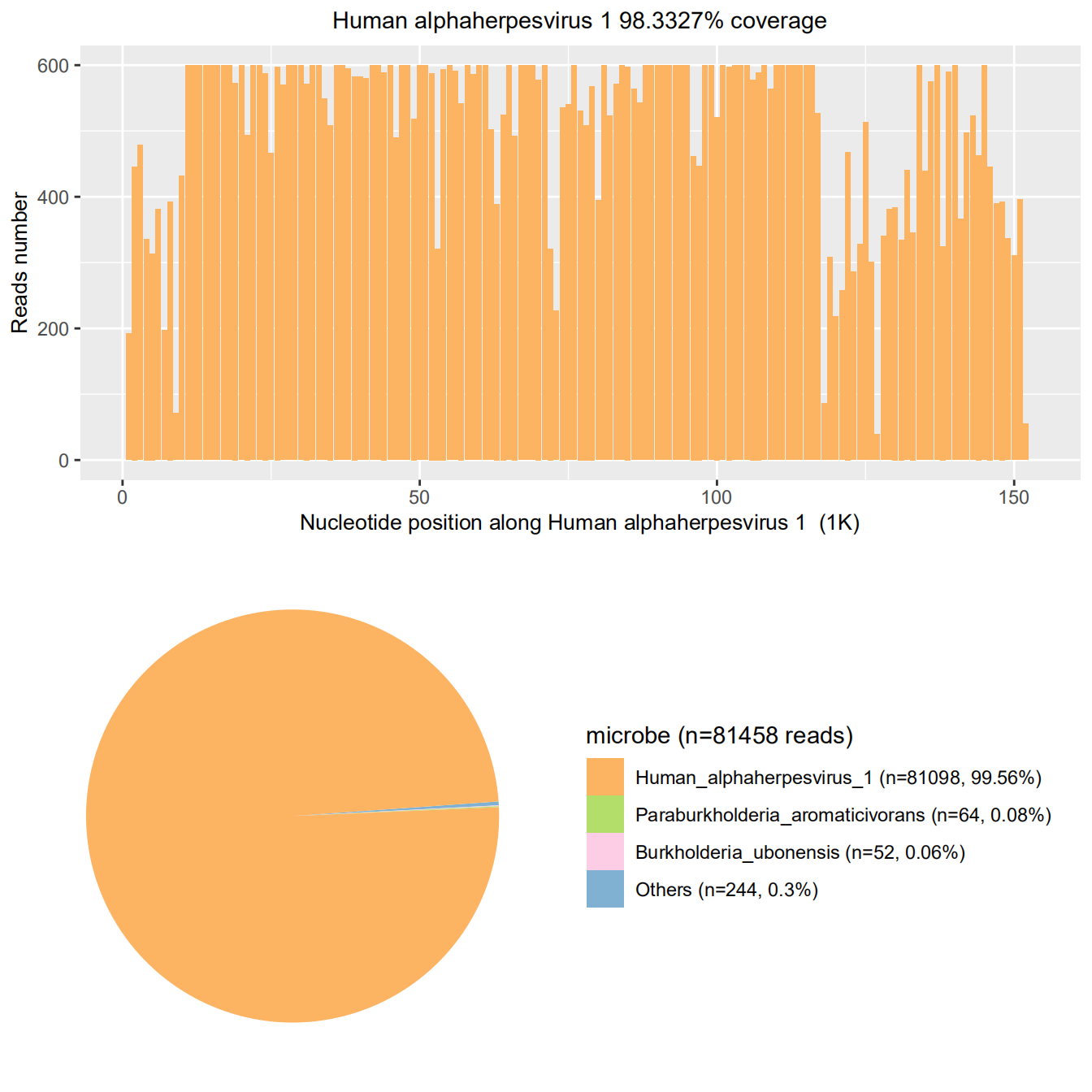


报告编号：6911179061


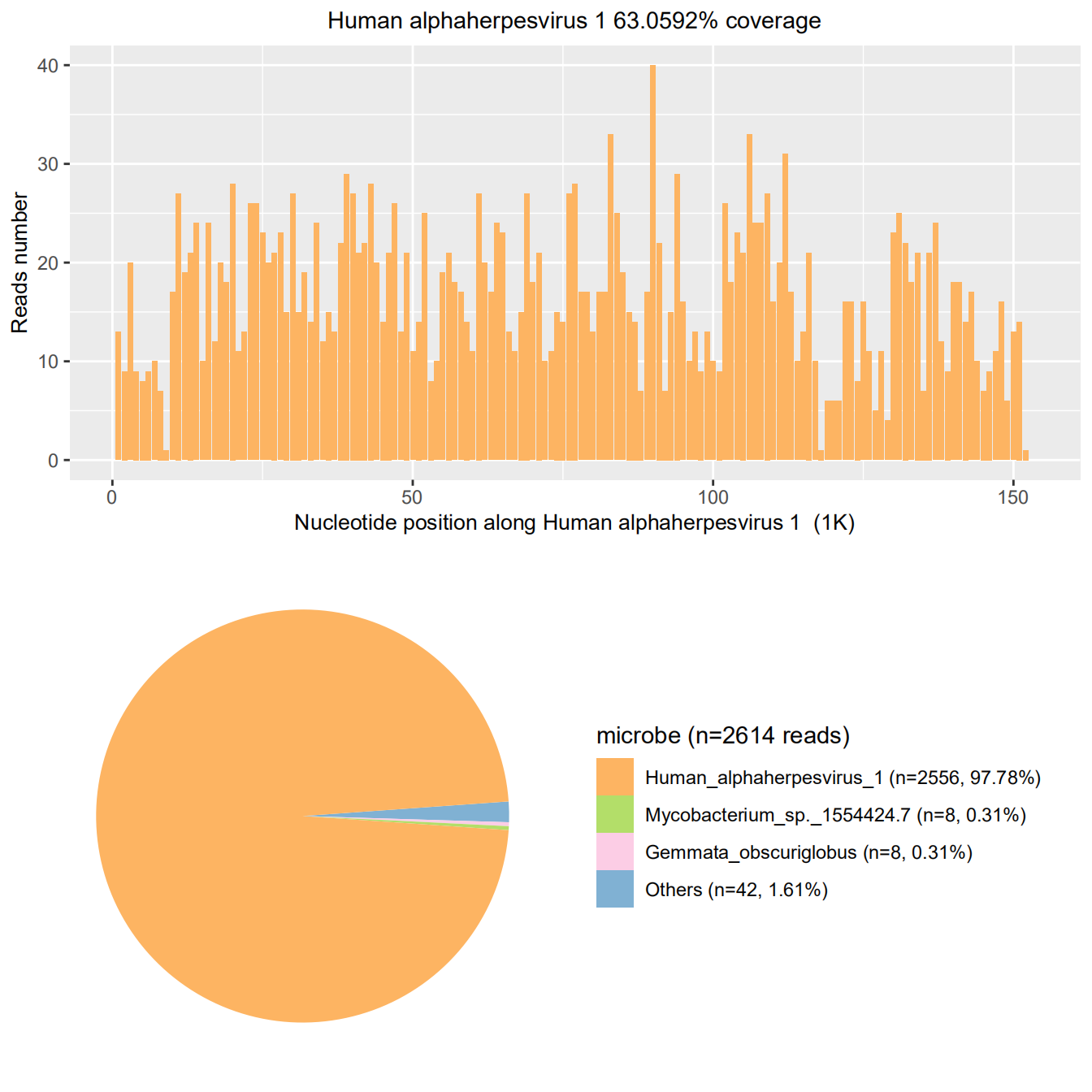

Supplement: Supplementary file 19 [file Data_Sheet_5.DOCX]
